# Restoration of Elbow Flexion with a Pedicled Latissimus Dorsi Myocutaneous Flap to a Brachial Plexus Injury at the Terminal Nerve Level

**DOI:** 10.1097/GOX.0000000000002472

**Published:** 2019-10-28

**Authors:** Takuya Kameda, Ejiri Soichi, Takeru Yokota, Shin-ichi Konno

**Affiliations:** From the * Department of Regional Medical Support for Orthopaedics, Fukushima Medical University, Fukushima, Japan; † Department of Orthopaedic Surgery, Iwaki City Medical Center, Fukushima, Japan.

## Abstract

A pedicled latissimus dorsi (LD) myocutaneous flap is a reliable reconstructive method for elbow flexion, though there are no reports regarding its application to a terminal nerve level injury of the brachial plexus. A 29-year-old man presented with dysfunction of elbow flexion, wrist extension, and finger extension. Physical examination and electromyography showed that the palsy was caused by an injury at the terminal nerve level of the brachial plexus without dysfunction of the axillary nerve. Bipolar transfer of LD for reconstruction of elbow flexion and subsequent tendon transfer for wrist and finger extension were performed. The final British Medical Research Council grade was 4 for elbow flexion, and active range of motion was 0/135. An injury at the terminal nerve level of the brachial plexus should be listed in the differential diagnosis of elbow flexion dysfunction even if shoulder function is intact, and a suitable reconstructive method for this atypical type of palsy could be bipolar transfer of a LD flap.

In cases of brachial plexus injury, it is generally difficult to reconstruct the function of the upper limb, and the rarity of a terminal nerve level injury of the brachial plexus (under 7% of all brachial plexus palsies^1^) makes it difficult to establish the standard protocol for functional reconstruction of this uncommon palsy.

Regarding the specific surgical method for reconstruction, especially for elbow flexion, bipolar transfer of a pedicled latissimus dorsi (LD) myocutaneous flap^2,3^ is one of the most prevalent procedures, though there are no reports of this method applied to a terminal nerve level injury of the brachial plexus.

A case of terminal nerve level injury of the brachial plexus without axillary nerve palsy treated with bipolar transfer of a LD myocutaneous flap for restoration of elbow flexion is described.

## CASE DETAILS

A 29-year-old man presented to our department with the chief complaints of dysfunction of elbow flexion, wrist extension, and finger extension. He was right-handed. One year earlier, his right limb was caught in machinery. The forearm was pulled forward by a conveyor belt, and his distal clavicle hit a metal pole. Since rupture of the right axiallary artery and pectoralis major muscle and fractures of the clavicle and scapula were diagnosed, revascularization and open reduction with internal fixation of the clavicle were performed immediately. However, the right upper limb palsy remained.

The skin scar was on the anterior side of the shoulder from the coracoid process to the axilla. The British Medical Research Council (BMRC) grading scale was 1 for biceps, 3 for triceps, 1 for brachioradialis, 1 for wrist extensors, 1 for finger extensors, and 5 for deltoid. Active range of motion (ROM) of the right shoulder was nearly full flexion and abduction (See Video 1 [online], which displays active motion of the shoulder, elbow, wrist, and finger before surgery). (See Video 2 [online], which displays active motion of the elbow, wrist, and fingers at one and a half years after the LD transfer).

**Video 1.** This video displays active motion of the shoulder, elbow, wrist, and finger before surgery.

**Video 2.** This video displays active motion of the elbow, wrist, and fingers at one and a half years after the LD transfer.

On MR imaging, no pseudomeningocele was observed. Biceps and brachialis showed denervation-re-innervation changes on electromyography.

Based on these findings, it was concluded that the cause of the palsy, which involved dysfunction of elbow flexion, as well as wrist and finger extension, was a terminal nerve level injury of the brachial plexus, especially the musculocutaneous nerve and part of the radial nerve. Since electromyography of the LD and the muscles innervated by the median nerve showed a normal pattern, transfer of LD for elbow flexion and subsequent tendon transfer for wrist and finger extension were scheduled. Intraoperative exploration using a nerve stimulator was not planned.

**Fig. 1.**
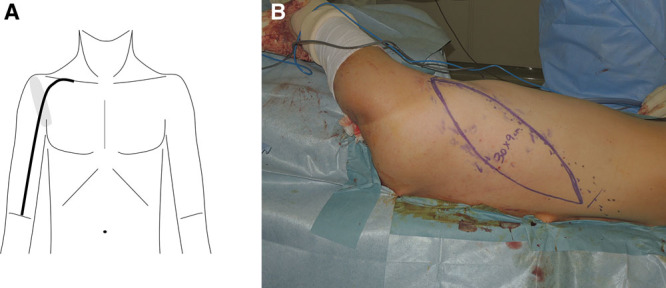
A, Schematic illustration of incision (bold line) for elbow flexion reconstruction with bipolar transfer of LD. Gray area is scar. B, Flap design.

**Fig. 2.**
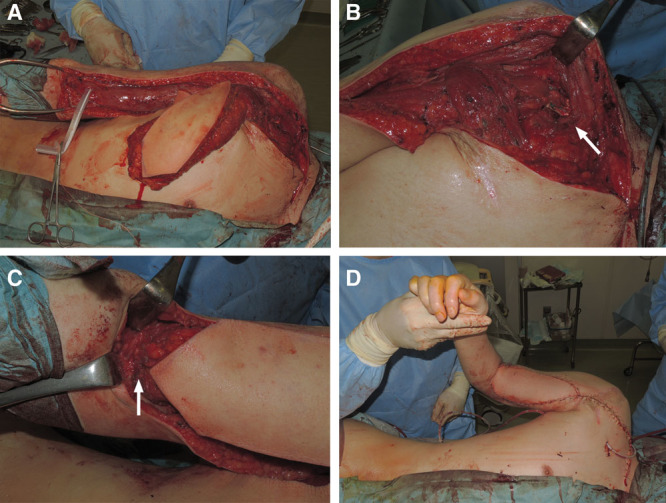
Passing the insertion of LD (humeral end) deep to the pectoralis major tendon (A), the humeral end of LD fixed to the coracoid process (B, arrow), the insertion of the biceps sutured to the origin of LD (C, arrow), and the final postoperative result (D).

Bipolar transfer of LD for reconstruction of elbow flexion was performed based on the method of Zancoli et al.^3^ With the patient in the left-lateral position, disinfection with povidone-iodine solution was carried out. After performing a right anterior longitudinal humerus incision, the coracoid process and insertion of the biceps were exposed. The LD flap was designed with a size of 9 cm × 30 cm and elevated as a pedicled myocutaneous flap (Fig. 1). After passing the insertion of LD (humeral end) deep to the pectoralis major tendon, this end of LD was fixed to the coracoid process with a suture anchor, and additional suture was performed for reinforcement. After trimming of the LD origin (caudal end), the flap length was adjusted so that the elbow joint remained spontaneously at 100 degrees with supination of the wrist. The insertion of the biceps was wrapped by the origin of LD and sutured. Four drains were placed to manage the dead spaces, and the incision was closed (Fig. 2). The elbow was fixed with a 2-mm Kirschner wire penetrating the ulna and humerus at 90 degrees, followed by splint fixation.

After 3 weeks, the Kirschner wire was removed, and active and passive ROM training for the elbow were started. Active elbow flexion was 130 degrees after 5 months.

Tendon transfer based on the method of Riordan^4^ with a slight modification was performed for the wrist and finger reconstruction as secondary surgery 5 months after primary surgery.

After splint fixation in the extension position of the wrist and finger for 3 weeks, active and passive ROM training began.

One and a half years after the primary surgery, the BMRC grade was 4 for elbow flexion, 4 for wrist extension, and 4 for finger extension. The active ROM of the right elbow was 0/135 (See Video 2 [online], which displays active motion of the elbow, wrist, and fingers at one and a half years after the LD transfer). The disabilities of the arm, shoulder, and hand score was 26.

## DISCUSSION

In this report, a case of brachial plexus injury at the terminal nerve level without obvious deficit of the axillary nerve was presented. The bipolar transfer of a myocutaneous LD flap resulted in a good outcome of reconstruction for the impairment of elbow flexion brought about by this atypical type of palsy.

**Fig. 3.**
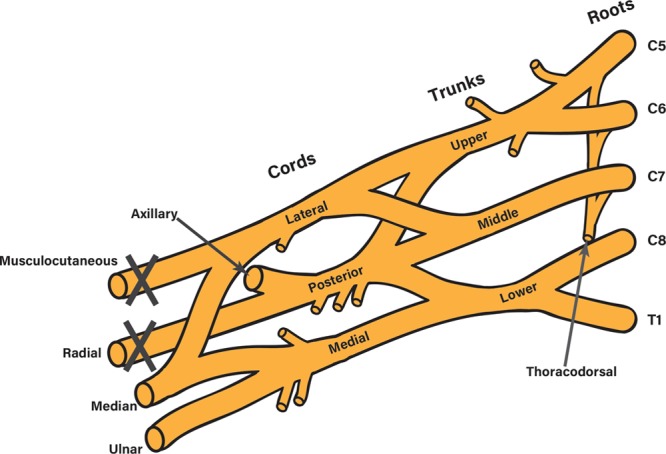
Schematic illustration of the suspected injured point (gray cross) in the brachial plexus.

A terminal nerve level injury of the brachial plexus without axillary nerve damage could be caused by trauma. In cases of brachial plexus palsy, infraclavicular localization, which consists of the cord or terminal nerve level, is seen in only 7% of cases.^1^ In cases of infraclavicular plexus injury, rupture of the axillary artery occurs in 50%.^5^ In the present case, the level of plexus injury was presumed to be infraclavicular from several clues: the situation of the injury, position of the scar, and rupture of the axillary artery. Moreover, the intactness of the axillary nerve with the impaired musculocutaneous nerve and radial nerve was the proof that the responsible level was not the root or trunk level (Fig. 3). However, in infraclavicular cases, the axillary nerve or the posterior cord is damaged in almost all cases.^5^ One outstanding and anomalistic feature of this case was good function of the shoulder with loss of elbow flexion. Thus, the possibility of infraclavicular brachial plexus injury cannot be ruled out even if shoulder function is intact.

A pedicled LD myocutaneous flap is effective for elbow flexion reconstruction in a case of terminal nerve level injury of the brachial plexus. This surgical method was introduced by Schottstaedt et al^2^ and summarized by Zancolli et al^3^, and several case series for the treatment of brachial plexus palsy have been reported.^6,7^ In this case, the function of LD was intact because of the injured level (Fig. 3), and notably, shoulder function was almost unimpaired. Thus, all joints (shoulder, elbow, wrist, and finger) of the injured limb finally worked. Terminal nerve brachial plexus injury could be a good indication for surgical reconstruction, especially for cases with unimpaired shoulder function.

In this report, it is not clear how this anomalistic palsy occurred. Because of some concomitant injuries, including fracture of shoulder girdle, neurological deficits, and rupture of the axially artery, type 3 scapulothoracic dissociation (SD) could have been one possibility.^8^ The injury mechanism, distraction with blunt force to the shoulder girdle, also suggests SD.^9^ Since several levels of neurological deficit can be observed in SD,^10^ SD with a direct mechanism can be the reason of this anomalistic palsy which is absence of elbow flexion with an acting shoulder.

A terminal nerve level injury of the brachial plexus without impaired shoulder function could be caused by trauma. Bipolar transfer of a myocutaneous LD flap could result in a satisfying functional outcome for elbow flexion in such injuries. The next question would be how to expand the indication for bipolar transfer of LD to elbow flexion reconstruction. Oberlin et al^11^ reported ulnar nerve transfer to the biceps motor nerve, and almost all cases showed BMRC grade 4 for elbow flexion within a 1-year preoperative delay.^12^ Integrated case series, a well-designed clinical trial, and a meta-analysis would be needed to answer this question.
